# Comparative transcriptome analysis reveals regulatory network and regulators associated with proanthocyanidin accumulation in persimmon

**DOI:** 10.1186/s12870-021-03133-z

**Published:** 2021-07-29

**Authors:** Qingyou Zheng, Wenxing Chen, Man Luo, Liqing Xu, Qinglin Zhang, Zhengrong Luo

**Affiliations:** Key Laboratory of Horticultural Plant Biology, Huazhong Agricultural University, Wuhan, 430070 Hubei China

**Keywords:** Persimmon, Regulatory network, Proanthocyanidin, Transcriptome, RNA-seq, Transcription factor, Co-expression networks

## Abstract

**Background:**

Proanthocyanidins (PAs) are important plant secondary metabolites that confer flavor, nutritional value, and resistance to pathogens. Persimmon is one of the PA richest crops. Mature fruits can be inedible because of the astringency caused by high PA levels and need to go through a de-astringency treatment before consumption. The molecular basis for PA accumulation is poorly known, particularly transcriptional regulators. We characterised three genotypes (‘Luotiantianshi’ (LT), ‘Mopanshi’ (MP), and ‘Youhou’ (YH)) with different PA accumulation patterns using an approach that combined PacBio full-length sequencing and Illumina-based RNA sequencing to build high-quality full-length transcriptomes. Additionally, we analysed transcriptome dynamics of the three genotypes (LT, MP, and YH) at four key fruit developmental stages.

**Results:**

A total of 96,463 transcripts were obtained. We identified 80,075 protein-coding sequences (CDSs), 71,137 simple sequence repeats (SSRs), and 27,845 long noncoding RNAs (lncRNAs). Pearson correlation coefficient (PCC), principal component analysis (PCA), and differentially expressed transcripts (DETs) analyses indicated that the four different developmental stages within a genotype exhibited similar transcriptome activities. A total of 2,164 transcripts specific to each fruit developmental stage were detected. The transcripts specific to early stages were attributed to phenylpropanoid and flavonoid biosynthesis. Co-expression network analyses revealed MEbrown and MEblue modules were strongly associated to PA accumulation. From these two modules, 20 hub TFs are potential regulators for PA accumulation. Among them, Cluster_78388 (SBP protein), Cluster_63454 (bZIP protein), and Cluster_66595 (MYB protein) appear to involve in the PA biosynthesis in Chinese genotypes.

**Conclusions:**

This is the first high-quality reference transcriptome for commercial persimmon. Our work provides insights into the molecular pathways underlying PA accumulation and enhances our global understanding of transcriptome dynamics throughout fruit development.

**Supplementary Information:**

The online version contains supplementary material available at 10.1186/s12870-021-03133-z.

## Background

Proanthocyanidins (PAs), also known as condensed tannins, are colourless phenolic compounds that confer quality and flavor to plant products such as wine, tea, and some berries [1, 2]. They play a critical role in plant defense mechanisms and are beneficial to human health because they provide protection against free radicals and cardiovascular diseases, among other health benefits [3]. PAs consist of flavan-3-ol monomers that are biosynthesised through the flavonoid branch of the phenylpropanoid pathway. The biochemical function of the flavonoid/PA pathway genes has been characterised in *Arabidopsis* [4, 5]. Two flavonoid reductases, anthocyanidin reductase (ANR) and leucoanthocyanidin reductase (LAR) catalyse the reactions of 2, 3-*cis*-flavan-3-ol and 2, 3-*trans*-flavan-3-ol synthesis, respectively [6, 7].

Persimmon (*Diospyros kaki* Thunb.; 2n = 6x = 90) is a woody tree and a widely cultivated crop that originates from East Asia. China is the biggest producer in the world and harbours more than 1,000 persimmon genotypes. Persimmon is particularly rich in PAs that constitute at least 1% of the fruit fresh weight [8]. The high PA concentration in persimmon fruit results in a strong sensation of dry or puckering mouth caused by the coagulation of oral proteins in response to PAs. Based on divergent properties of astringency removal in fruit, persimmon genotypes are classified into two types: pollination-constant nonastringent (PCNA) and non-PCNA [9]. Fruit of the PCNA type loses astringency naturally and becomes edible at maturity while the non-PCNA fruit maintains its strong astringent taste when mature and is inedible. The Chinese PCNA (C-PCNA) and the Japanese PCNA (J-PCNA) persimmon derive from two types of spontaneous mutations and have different PA regulation mechanisms. J-PCNA persimmon is determined by a recessive allele *ast*, and C-PCNA genotype is determined by a dominant allele *CPCNA* [10–12]. Nishiyama et al. has delimited the *AST* gene locus to 915-kb region, but the underlying gene is still not identified [13].

Recent studies have shown that transcription factors (TFs) play a critical role in controlling gene expression related to the flavonoid pathway through the ternary complex [14, 15]. In *Arabidopsis*, MYB factor TT2 controls PA accumulation by interacting with the bHLH protein TT8 and the WD-40 factor TTG1 [16]. PA regulation via the MBW complex has also been identified in apple, strawberry, and poplar [17, 18]. In persimmon, two MYB TFs, DkMYB2 and DkMYB4, are involved in PA biosynthesis by forming the MBW complex with DkMYC1 (bHLH) and DkWDR1 (WD-40) [19–22]. Several TFs are involved in flavonoid biosynthesis by regulating the MBW complex. In *Arabidopsis*, TT1 (WIP domain-containing zinc-finger protein), TTG2 (WRKY family protein), and TT16 (MADS domain protein) regulate PA biosynthesis by interacting with the MBW complex (TT2, TT8, and TTG1) in seed coat [23–25]. MYBL2 acts as a negative regulator in anthocyanin biosynthesis by inhibiting the MBW complex [26, 27]. MdMAC52 regulates anthocyanin and PA accumulation by controlling the expression of *MdMYB9/11* in the MBW complex [28]. In *Arabidopsis,* E3 ligase COP1/SPA interacting with the MYB TF PAP1 and PAP2 control anthocyanin levels [29]. The transcriptional regulation of flavonoid/PA biosynthesis is complex as numerous TFs are involved in the biosynthetic process [30]. Little is known about other regulators and the regulatory networks associated with PA accumulation in persimmon.

Transcriptomic profiling is widely used to assess species genetic diversity and allows to investigate the molecular basis of specific traits of interest [31–35]. Illumina-based RNA sequencing is a powerful tool for quantifying gene expression, but the computational challenges in de novo assembly using short-reads limit its application in organisms without a reference genome [36]. The full-length transcriptome generated by Pacific BioSciences (PacBio) has been commonly used for organisms lacking a complete reference genome because the resulting transcriptome can be used as a reference without the genome. The PacBio Iso-seq is a single-molecule real-time sequencing with read lengths up to 20 kb. It yields full-length transcripts from 3’ to 5’ end eliminating the need of assembly [37]. The integration of Illumina RNA-seq with PacBio Iso-seq has been widely applied to quantify transcript expression throughout plant development [38–41]. In this study we integrated Illumina RNA-seq and PacBio Iso-seq to investigate the regulators and the regulatory networks involved in PA accumulation in three persimmon genotypes with different PAs accumulation patterns: LT (C-PCNA), MP (non-PCNA), and YH (J-PCNA). We constructed a reference transcriptome and analysed transcriptome dynamics during four fruit developmental stages.

## Results

### Quantitation of PA content in three persimmon genotypes

**Fig. 1:**
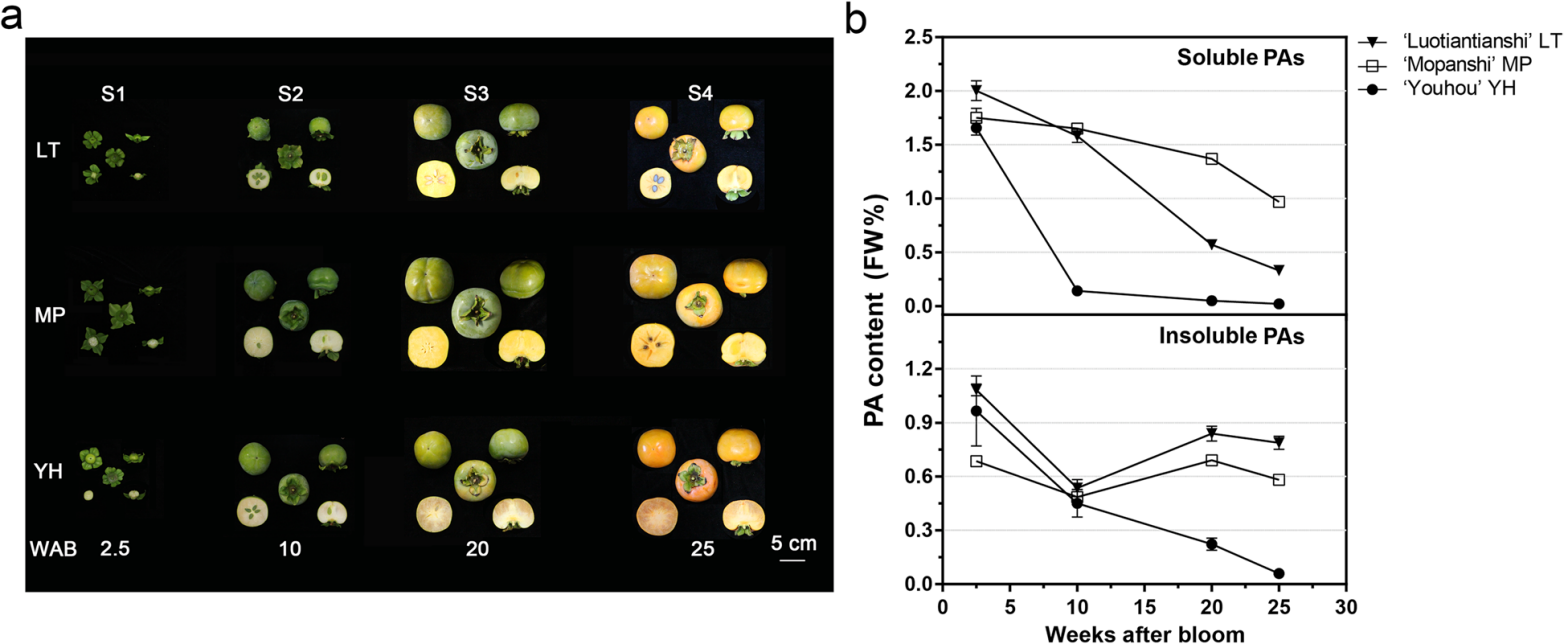
Fruit morphology and PA content at 2.5, 10, 20, 25 weeks after bloom in LT, MP, and YH. **a** The fruit morphology of the three genotypes at four stages. **b** The changes of fruit PA content at four stages in the three genotypes. Each value represents a mean ± SD of three independent biological replicates

It takes about 25 weeks for the persimmon fruit to reach maturity. Figure 1 a shows the morphological features of the three genotypes LT, YH, and MP at four different stages: 2.5 (S1), 10 (S2), 20 (S3), 25 (S4) weeks after bloom. From S1 to S3, fruit size kept rising. From S3 to S4, fruit maturity was reached and peel colour changed from green to orange. The three genotypes showed distinct PA accumulation patterns throughout fruit development (Fig. 1b). All the three genotypes started with high soluble-PA content at S1, ranging from 2.0 FW% for LT to 1.7 FW% for YH. Levels of soluble PA differed significantly in the subsequent fruit developmental stages. Soluble PA for YH decreased rapidly to 0.1 FW% at S2 while soluble PA levels for LT (1.6 FW%) and MP (1.7 FW%) remained high. Soluble PA levels in LT decreased notably (0.3 FW%) at S4, when the fruit became edible without treatment. Conversely, soluble PA levels in MP were still over 1.0 FW% at that time. The insoluble PA levels for Chinese genotypes decreased from S1 to S2 and increased from S2 to S4. The insoluble PA for Japanese persimmon YH, on the other hand, showed a continuous decrease throughout fruit development.

### Construction of a reference transcriptome with PacBio Iso-seq

A total of 694,499 and 623,048 reads of inserts (ROIs) were obtained. The average read length was 2,558 bp and 2,777 bp for 0–5 Kb and 4.5–10 Kb libraries, respectively (Additional file 1: Table S1). Of these, 38.54% and 49.86% were full-length non-chimeric reads, 53.13% and 41.48% were non-full-length reads. A total of 93,896 and 119,343 high-quality (hq) isoforms were generated for each library. The distribution length of hq isoforms was consistent with the size of the two libraries (Additional file 2: Figure S1). The redundant hq isoforms were removed and further corrected using paired-end reads. After correction, we obtained 96,463 transcripts with a N50 value of 5,326 bp and 41.09% GC content (Table 1). Benchmarking universal single-copy orthologs (BUSCO) analysis indicated 92% completeness (Additional file 2: Figure S2a). Collectively, the 96,463 full-length transcripts with high quality and completeness were considered as reference transcriptome of the hexaploid persimmon.

**Table 1:** Summary of reference transcriptome generated by PacBio Iso-seq

	Reference transcriptome
**Statistics of the final reference transcripts**	
Total_number	96,463
N50 length (bp)	5,326
N90 length (bp)	1,289
Max_length (bp)	13,843
Min_length (bp)	290
Sequence_GC (%)	0.4109
**Annotation**	
Nr	88,951 (92.21%)
Nt	78,865 (81.76%)
Swissprot	69,805 (72.36%)
KEGG	75,986 (78.77%)
KOG	75,347 (78.11%)
Pfam	72,158 (74.8%)
GO	46,625 (48.33%)
Intersection	30,264 (31.37%)
Overall	91,228 (94.57%)

### Functional annotation and prediction of CDS, SSR, and lncRNA

Transcripts were aligned to seven databases for annotation: NCBI non-redundant protein (NR), NCBI non-redundant nucleotides (NT), SwissProt, Kyoto encyclopedia of genes and genomes (KEGG), EuKaryotic orthologous groups (KOG), Protein family (Pfam), and Gene ontology (GO). A total of 91,228 transcripts (94.57%) were annotated with at least one database, and 30,264 transcripts (31.37%) were annotated with all the seven databases (Additional file 2: Figure S2b). The transcript number annotated by NR was the highest (88,951, 92.21%), while the minimum of 46,625 (48.33%) transcripts were annotated with GO database (Table 1). Of the reference transcripts, 80,075 CDSs were identified. Sequence length ranged from 297 bp to 8,622 bp and 92.8% of the sequences were 400—3000 bp long. N50 value was 1,563 bp and GC content was 45.08% (Additional file 2: Figure S2c and Additional file 3: Table S2). We found 41,344 SSR-containing sequences and 71,137 SSRs with tandem repeat motifs of 1–6 bp in length. Overall, 23,016 (32.4%) were mono-nucleotide, 33,189 (46.7%) were di-nucleotide, 12,241 (17.2%) were tri-nucleotide, 916 (1.3%) were quad-nucleotide, 832 (1.2%) were penta-nucleotide, and 943 (1.3%) were hexa-nucleotide. Among them, 8,096 SSRs were present in compound formation (Additional file 3: Table S2). A total of 27,845 transcripts were identified as lncRNAs. Sequence length ranged from 290 to 10,489 bp. N50 value was 5,450 bp and GC content was 39.23% (Additional file 2: Figure S2d and Additional file 3: Table S2).

### Global transcriptome analyses of three genotypes with Illumina RNA-seq

**Fig. 2:**
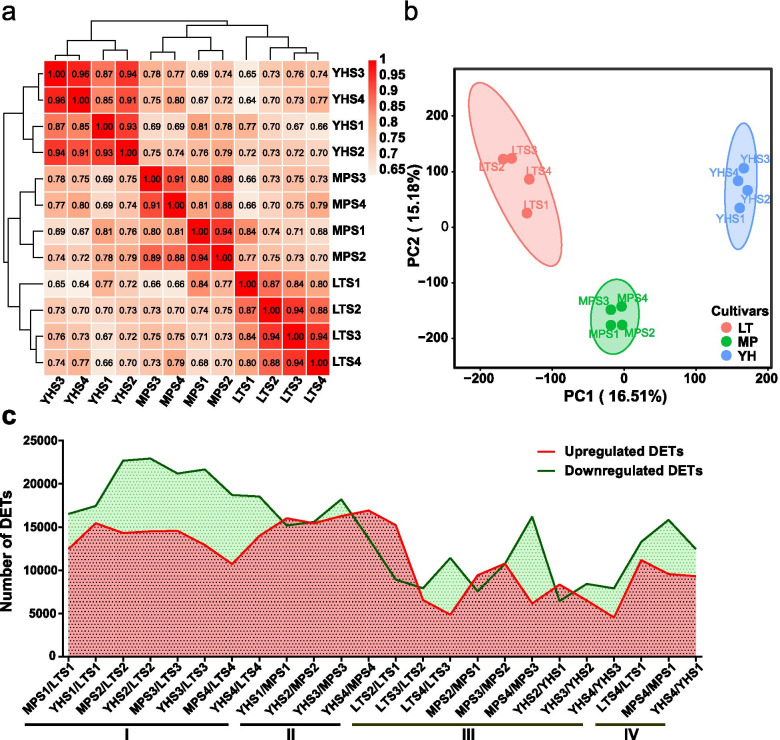
Global transcriptome profiling of LT, MP, and YH at four developmental stages. **a** PCC analysis of RNA-seq data of three genotypes at four stages. **b** PCA of RNA-seq data of three genotypes at four stages. **c** DETs identified from pairwise comparisons of LT, MP, and YH at four stages. I, Pairwise comparisons of LT with MP and YH at each stage; II, Pairwise comparisons between YH and MP at each stage; III, Pairwise comparisons between the adjacent stages within a genotype; IV, Pairwise comparisons between the S1 and S4 stages of the three genotypes

An average of 42.86 million clean reads were generated and mapped to the reference transcriptome constructed above with an average mapping ratio of 77.41%. A total of 72,100 transcripts (FPKM ≥ 0.5) were detected as expressed transcripts. The proportion of non-expressing (FPKM < 0.5), low-expressing (0.5 ≤ FPKM < 10), and high-expressing (FPKM ≥ 10) transcripts of the three genotypes at each stage were similar, with the exception of the non-expressing transcripts at S2, S3, and S4 stages in LT, which were lower (Additional file 2: Figure S3). PCC values were higher between the fruit developmental stages within a genotype than between genotypes, which indicates similar gene events within genotypes (Fig. 2a). Consistent with these results, the four developmental stages of the same genotype were tightly grouped, and the three genotypes separated from each other in the PCA plot (Fig. 2b).

DET pairwise comparison between the three genotypes at each fruit developmental stage showed the biggest difference in the number of up- and down-regulated DETs in MPS4/MPS3 (10,033 more down-regulated DETs in MPS4), while the maximal DETs number (the sum of up- and down-regulated transcripts) were observed in YHS2/LTS2 (37,443 total DETs) (Fig. 2c). The LT genotype harboured more up-regulated transcripts compared with the other two genotypes at each stage (Cluster I). A maximum of 22,937 up-regulated transcripts were obtained for LTS2 compared to YHS2, and a minimum of 16,517 up-regulated transcripts in LTS1 compared to MPS1. The large number of DET specific to LT suggests that unique developmental programs might be occurring in this genotype. And the number of total DETs (the sum of the up and down-regulated transcripts) identified from the comparisons across the genotypes at each stage (Cluster I and II) were significantly higher than that identified from the adjacent stages within the same genotype (Cluster III and IV). The difference in DET numbers between the three genotypes is congruent with the results obtained with the PCA and PCC analyses (shown above, Fig. 2a and b), and suggests that more relevant the transcriptome activities within a genotype rather than the identical developmental stage between the genotypes.

### Identification of transcript sets specific to a particular developmental stage

**Fig. 3:**
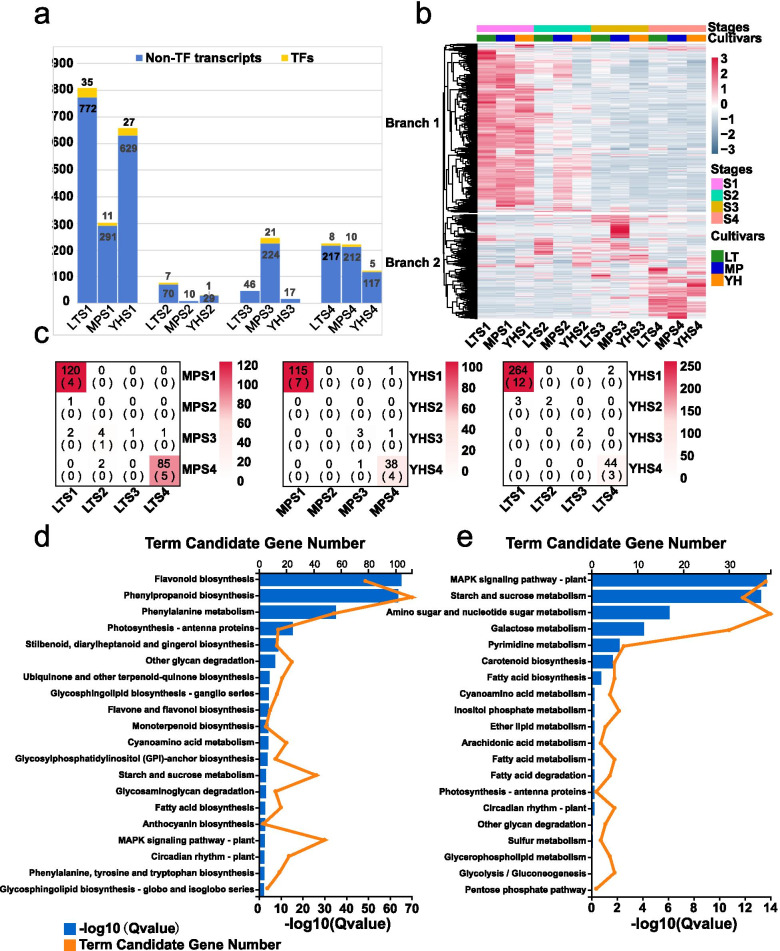
Identification of stage-specific transcripts at each developmental stage for LT, MP, and YH. **a** Bar graph showing the number of SS transcripts and TFs for the three genotypes at four stages. **b** Heatmap showing the expression profile of SS transcripts of the three genotypes at four stages. **c** Heatmap showing the number of the overlapping SS transcripts of the three genotypes at each stage. Boxes indicates the numbers of the overlapping transcripts (including TFs), and the TF numbers are shown in the parentheses. **d** The KEGG enrichment analysis of S1- and (**e**) S4-specific transcripts commonly identified in the three genotypes. The top 20 enriched KEGG terms are displayed

The transcripts that express specially at a certain stage were defined as stage-specific (SS) transcripts. We identified 1,155, 779, and 825 SS transcripts for the LT, MP, and YH genotypes respectively (Additional file 4: Tables S3, S4, and S5). The number of SS transcripts between the two PCNA genotypes were similar across the four developmental stages. SS transcripts were most abundant at S1 with 807 for LT and 656 for YH, followed by S4 with 225 for LT and 122 for YH. SS transcripts were lowest at S3: LT (46) and YH (17), and none for TF. The number of SS transcripts in the MP genotype was different from two PCNA genotypes (LT and YH): S1 (302), S2 (10), S3 (245), and S4 (222) (Fig. 3a). The total 2,164 SS transcripts that were hierarchically clustered and fell into two branches in the heatmap (Fig. 3b). Branch 1, with 1,339 transcripts, consisted predominantly of S1-specific transcripts. A significantly overlap SS transcripts was observed at S1 among the three genotypes and at S4 as well. LTS1 and YHS1 showed the highest overlap in SS transcripts (264, including 12 TFs), which accounted for 42% of the total SS transcripts in YH. LTS4 and MPS4 shared 85 (including 5 TFs) common transcripts, corresponding to 40.5% of the total SS transcripts in MPS4. The number of overlapping SS transcripts between LTS4 and MPS4 (85, including 5 TFs) was significantly higher than that between LTS4 and YHS4 (44, including 3 TFs) (Fig. 3c).

Substantial common SS transcripts were observed at S1 and S4 stages, then those SS transcript sets for S1 (branch 1, 1,339 transcripts) and S4 stage (427 transcripts) were mapped to the KEGG database. The most significantly enriched KEGG terms for S1 were associated with flavonoid biosynthesis, phenylpropanoid biosynthesis, and phenylalanine metabolism (Fig. 3d). Metabolic visualization with MapMan also assigned S1-specific transcripts to the phenylpropanoid/flavonoid pathway. A hundred and eight transcripts representing thirteen biosynthetic genes were attributed to the phenylpropanoid pathway, and most of these transcripts showed high expression levels in LTS1 (Additional file 2: Figure S4). S4-specific transcripts were attributed to starch and sucrose metabolism, galactose metabolism, carotenoid biosynthesis, and fatty acid biosynthesis. These results are congruent with the fact that S4 is the fruit ripening stage when edulcoration of the taste and colouration of fruit peel occur (Fig. 3e).

### Weighted gene co-expression network analysis (WGCNA)

**Fig. 4:**
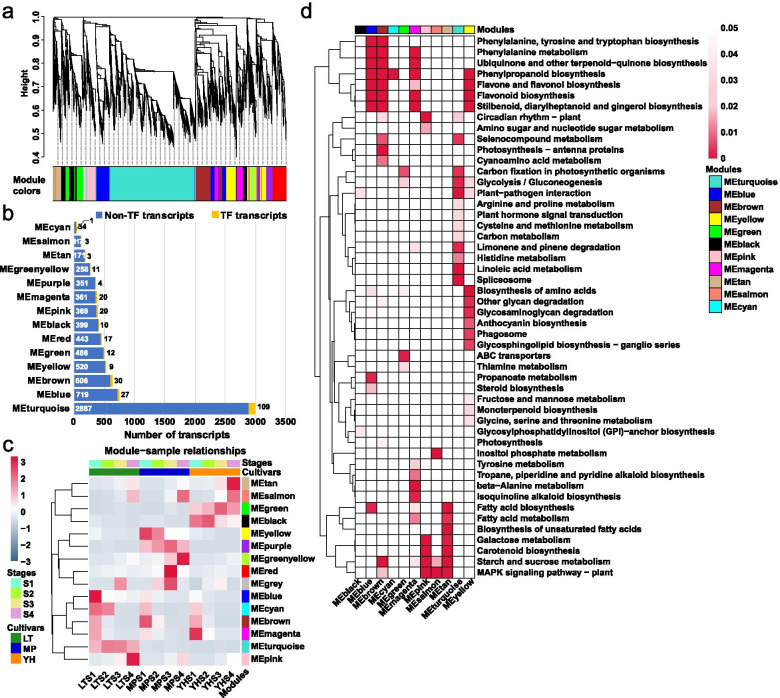
Weighted gene co-expression network analysis of high-variance transcripts in LT, MP, and YH genotypes during fruit development. **a** Hierarchical clustering tree showing the co-expression modules identified by WGCNA. Each module is labeled with different colour, and each leaf in the tree corresponds to one transcript. **b** Numbers of the TFs and non-TF transcripts in each module. **c** Module eigengene expression. The colour of each cell at the row–column intersection indicates the expression level of the module eigengene. **d** KEGG enrichment analysis of the WGCNA modules. All the KEGG terms with Qvalue < 0.05 are displayed

The WGCNA was performed to reveal the interconnected gene sets that were associated with PA accumulation. Transcripts were grouped into fourteen co-expression modules (Fig. 4a). Transcript number within each module was variable. The MEturquoise module had the highest number of transcripts (2,996, including 109 TFs) while MEcyan had the lowest number (35, including 1 TF) (Fig. 4b and Additional file 5: Table S6). Transcripts in the MEturquoise module showed higher expression levels in the LT genotype. MEgreen and MEblack modules showed high expression levels in the YH genotype, and the MEpurple module had the highest expression levels in the MP genotype. MEblue, MEcyan, MEbrown, and MEmagenta exhibited high expression levels in the early developmental stages in all three genotypes. In contrast, MEtan, MEpink, and MEsalmon modules were expressed preferentially in late fruit developmental stages (Fig. 4c). KEGG mapping of the modules expressed in the late stages indicate that these were related to fatty acid biosynthesis, fatty acid metabolism, biosynthesis of unsaturated fatty acids, galactose metabolism, carotenoid biosynthesis, and starch and sucrose metabolism. These results imply that fatty acids, galactose, carotenoid, starch, and sucrose are synthesised during the late stages of fruit maturation. KEGG mapping of MEblue and MEbrown modules showed that they were involved in phenylalanine, tyrosine and tryptophan biosynthesis, phenylalanine metabolism, ubiquinone and other terpenoid–quinone biosynthesis, phenylpropanoid biosynthesis, flavone and flavonol biosynthesis, flavonoid biosynthesis, and stilbenoid, diarylheptanoid and gingerol biosynthesis (Fig. 4d). Such results strongly suggest an active biosynthesis of phenylpropanoid/flavonoid in all three genotypes at early developmental stages.

### Characterization of regulatory networks associated with PA accumulation

**Fig. 5:**
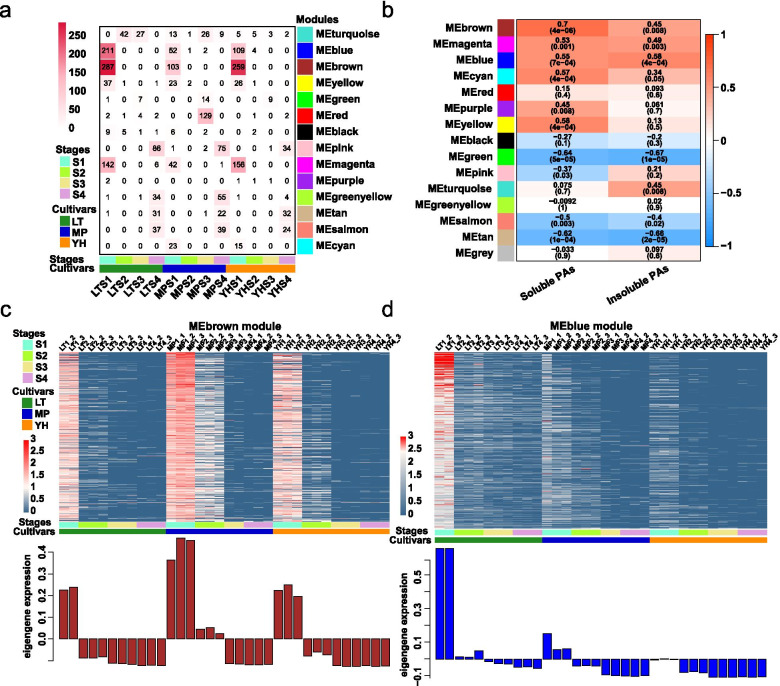
Co-expression modules analysis. **a** Relationships of the SS gene sets with the co-expression modules identified by WGCNA. Columns represent the SS gene sets of the three genotypes, and rows represent the co-expression modules. The number in the box indicates the number of overlapping transcripts (including TFs) between the SS gene set and co-expression module. **b** Heatmap showing the module-sample associations. Each row corresponds to a co-expression module, and the two columns correspond to the soluble-PA content and insoluble-PA content, respectively. The colour of each cell at the row-column intersection indicates the correlation coefficient between the module and PA content. **c** Heatmap showing the expression profiles of the transcripts within the MEbrown module and (**d**) MEblue module. The corresponding bar graphs showing the eigengene expression levels at four stages and replicates in the three genotypes

We previously identified SS transcripts for each stage, and we found that the KEGG terms enriched in the S1–specific transcripts showed high similarity with the MEbrown and MEblue modules. Therefore, the overlapping transcripts between the SS transcripts and WGCNA modules were examined. Our results showed that the MEbrown module shared the highest number of overlapping transcripts with LTS1 (287), YHS1 (259), and MPS1 (103), followed by the MEblue (211 for LTS1, 109 for YHS1, and 52 for MPS1) and MEmagenta (142 for LTS1, 156 for YHS1, and 42 for MPS1) modules (Fig. 5a). MEbrown exhibited the highest correlation with soluble PAs (0.7) and MEblue showed the closest relationship with insoluble PAs (0.58) (Fig. 5b). The expression levels of the MEbrown transcripts were significantly higher in LTS1, MPS1/S2, and YHS1 samples (Fig. 5c). The bar graph showed that opposite expression pattern was observed, with higher transcript levels at S1 of the three genotypes and S2 of MP. Likewise, higher expression levels were generally detected at S1 compared to S2, S3, and S4 in all three genotypes in MEblue module (Fig. 5d). The KEGG analyses showed those two modules were related to phenylpropanoid/flavonoid pathway (Additional file 2: Figure S5).

### Identification of hub TFs involved in PA biosynthesis during early developmental stages

**Fig. 6:**
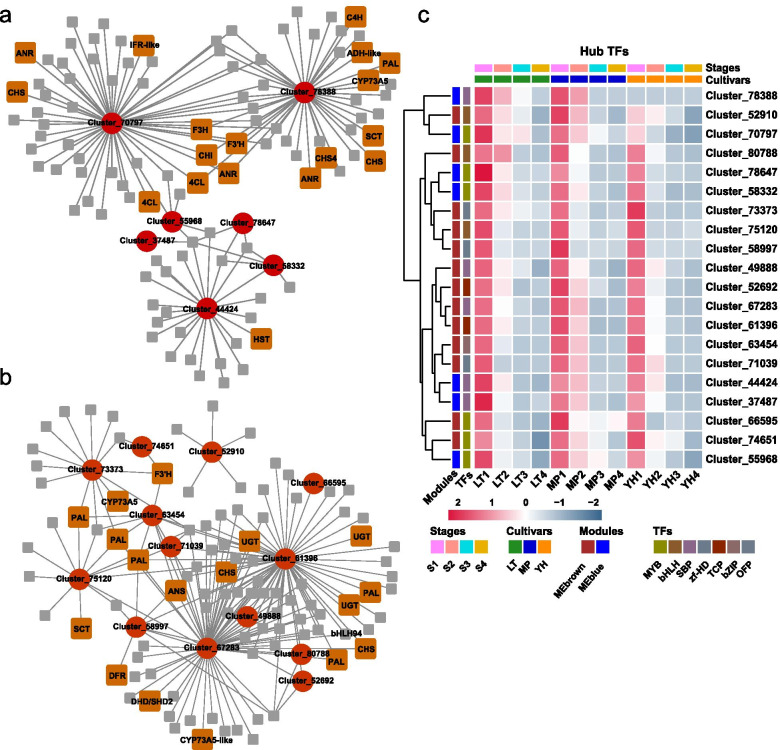
Regulatory network analyses of PA-related modules. **a** The transcriptional regulatory network of the candidate TFs in MEblue module and (**b**) MEbrown module. Each red-circle represents the candidate TF (node), and each brown-square represents a phenylpropanoid/flavonoid pathway synthetic transcript (edge). **c** Heatmap showing the expression patterns of the candidate hub TFs

We identified 27 TFs in the MEblue module and 30 TF genes in the MEbrown module (Additional file 6: Table S7). The TFs with high intramodular connectivity and gene significance (GS) were defined as candidate TFs. Nine TFs from MEblue and twenty from MEbrown modules were selected as candidate regulators. Using the candidate TFs as baits, all the transcripts related to them were extracted. Seven hub TFs in MEblue and thirteen in MEbrown co-expression network with strong connectivity with their corresponding edges (phenylpropanoid/flavonoid pathway genes) were identified (Fig. 6a and b). Expression patterns of the 20 hub TFs are shown in Fig. 6c. From the MEblue module, Cluster_78388 (SQUAMOSA promoter-binding protein (SBP box)), harbouring the most edges related to the phenylpropanoid/flavonoid pathway, was highly expressed at S1 and S2 in the LT and MP genotypes, but was not expressed in the YH genotype. Cluster_70797 (MYB TF), had the second most edges, showing high expression levels at S1 and S2 in all three genotypes. In the MEbrown module, Cluster_67283 (SBP-box TF), Cluster_61396 (TCP protein), and Cluster_63454 (bZIP TF) harboured the most phenylpropanoid/flavonoid associated genes. Among them, Cluster_63454 maintained particularly high expression levels until S2 in the MP genotype. In addition, Cluster_66595 (MYB protein) was continuously expressed in MP and was also defined as a putative PA-associated TF.

## Discussion

### PacBio Iso-seq provides high-quality reference transcriptome

Persimmon is an important crop in East Asia and has been widely cultivated in China, Japan, and Korea. Since persimmon is hexaploid and a reliable reference genome has not yet available, the molecular mechanisms underlying important agronomic traits are poorly understood. We generated a full-length reference transcriptome of the hexaploid persimmon LT genotype using both PacBio Iso-seq and Illumina-based RNA-seq. The reference transcriptome comprised 96,463 transcripts, with maximum read length of 13,843 bp and a N50 value of 5,326 bp. About 95% transcripts were annotated with at least one database (Table 1). The BUSCO analysis showed high completeness of the reference transcriptome (92%) [42]. These results are a major improvement from previous studies that used other sequencing methods such as 454 sequencing in which a total of 83,898 genes were identified using de novo assembly and only 65.2% of them were annotated in the NR database [43]. Chen et al. (2017) performed shotgun sequencing and 135,999 genes were de novo assembled from short reads of the ‘Eshi 1’ genotype, only 63.8% of which were annotated [44]. The transcriptome of non-PCNA ‘Taiten’ was de novo assembled, and only 37.5% of contigs got annotated by the NR database [45]. Compared with 95% transcripts annotated in this work, the lower annotation ratio of the genes in the previous study may ascribe to the substantial artifacts produced from de novo assembly. Moreover, PacBio sequencing yielded a full-length transcriptome with a N50 value of 5,326 bp, which is longer than the transcripts assembled from short reads of ‘Eshi 1’ (2,071 bp) and ‘Taiten’ (726 bp). The PacBio Iso-Seq capturing the full-length transcript without assembly overcomes the difficulties posed by the short-read data in comparison of the de novo assembled transcriptome from short reads. The high quality, full-length transcriptome presented in this study can be used as reference transcriptome for future research. Furthermore, full-length transcripts enable prediction of alternative splicing and polyadenylation that are responsible for various biological processes [46–48].

### Stage-specific transcripts enlighten the PA biosynthesis pathways at the early stages

Former studies have identified the compartment-specific genes that are expressed specially at each compartment of maize kernel. Zhan et al. (2015) identified compartment-specific genes to understand the difference in gene expression and the biological processes in each subregion [49]. Garg (2017) identified the SS genes at each stage during seed development in chickpea. These SS transcripts reveal the specific biological processes undergoing at that stage. As such cell cycle and growth processes in early stages; cell wall, lipid metabolism, secondary metabolism, and protein synthesis in mid-stages; and abiotic stress, transcription, and protein folding in late stages during seed development [50]. The KEGG and MapMan analyses showed that the SS transcripts in branch 1 expressed at S1 were closely associated with flavonoid regulation (Fig. 3d). Which implies that PA biosynthesis process are ongoing actively during early stages in all the three genotypes. These results are congruent with previous research and confirm differential gene expression throughout fruit development [9, 51]. In addition, the S4-specific transcripts obtained for all three genotypes attributed to fatty acid, carotenoid, galactose, starch and sucrose biosynthesis are congruent with fruit maturation processes characteristic of this developmental stage (Fig. 3e). The high number of common SS transcripts between LTS1-YHS1 suggests greater similarity in PA accumulation pattern within the PCNA type during early developmental stages. LT and MP genotypes showed a higher number of SS transcripts during S4. Likewise, shared SS transcripts between LTS4-MPS4 indicate similar transcriptomic activity in Chinese persimmon during late developmental stages (Fig. 3c), which reflects in the specific polymerization or biochemical reaction of the 2,3-*cis*/*trans*-flavan-3-ols is ongoing at S4, and could explain the coagulation effect (conversion of soluble PAs into insoluble PAs) specific to the Chinese genotypes [52–54].

### Characterization of hub TFs involved in PA biosynthesis

The biosynthetic genes involved in the phenylpropanoid/flavonoid pathway [14–16, 30, 55] and the biosynthesis of PA monomers 2,3-*cis*-flavan-3-ols and 2,3-*trans*-flavan-3-ols have been well studied, more studies focus on the regulation of those pathway genes in transcriptional level [6, 7]. Previous research has found that the co-expression of the Pink module is associated with anthocyanin synthesis in apple, and that the two genes with the highest GS in the Pink module are important regulators [31]. Li et al. (2020) identified MYB8 as the key flavonoid-associated regulator which is the hub TFs of the MEdarkslateblue module showing highest correlation with flavonol biosynthesis in crabapple [56]. In this study, we constructed gene regulatory networks by examining 8,000 high-variation transcripts using WGCNA and effectively characterised co-expression regulatory networks and potential gene-TF regulations. Those 8,000 transcripts for WGCNA analysis included a hundred and thirteen phenylpropanoid pathway transcripts (79.6% total phenylpropanoid-related transcripts). We characterised two modules MEblue and MEbrown that show high correlation with PA biosynthesis. Gene expression in these two modules showed contrasting expression patterns at key stages.

Moreover, these two modules exhibited the highest number of common transcripts with S1-specific transcript sets. Two MYB TFs, DkMYB2 and DkMYB4, formerly characterised as PA-associated regulators in persimmon, were identified in these two modules [19, 20]. Thereby, these two co-expression modules were here considered as PA-associated gene sets. Seven and thirteen TFs were identified as potential gene expression regulators in the MEblue and the MEbrown modules respectively (Fig. 6a and b). Recent studies have shown that the SBP and TCP families play an important role in the biosynthesis of secondary metabolites in plants. For example, the SQUAMOSA subfamily of MADS box gene *VmTDR4*, and the target gene of the SBP protein CNR, are involved in the activation of anthocyanin biosynthesis in bilberry [57]. A SBP-like gene *SPL9*, down-regulates anthocyanin biosynthesis by destabilizing the MBW complex in *Arabidopsis* [58]. TCP3 could interact with the R2R3-MYB of the MBW complex, and promote anthocyanin and PA biosynthesis via stabilizing the MBW complex [59]. In this study, Cluster_78388 (SBP TF) in MEblue showed a strong connection with thirteen edge genes. Cluster_67283 (SBP TF) and Cluster_61396 (TCP) harboured the highest number of transcripts in the MEbrown module. Therefore, the two SBP proteins Cluster_78388, Cluster_67283, and one TCP protein Cluster_61396 are potential PA regulators. The high expression levels of Cluster_78388 in the LT and MP genotypes reveal a potential function in PA accumulation in the Chinese genotypes. Similarly, the continuous expression of cluster_63454 (bZIP) during S1 and S2 in non-PCNA persimmon indicates that it may play a specific role in PA accumulation in non-PCNA genotypes. The expression levels of Cluster_66595 (MYB) throughout the four stages in the MP genotype also suggests a key regulator in PA accumulation. We conclude that Cluster_78388 was the potential specific regulator in Chinese genotypes, while Cluster_63454 and Cluster_66595 were speculated to involve in continuous accumulation of PAs in non-PCNA genotypes. In addition, those edges showing connection with hub TFs function as the putative target genes. Overall, our analyses identify the PA-associated regulatory networks and hub TFs, which provide the crucial candidate genes for core germplasm construction via genetic manipulation.

## Conclusions

Here we present the first high-quality reference transcriptome for persimmon, which provides the base for further research on the molecular basis of the metabolic pathways involved in fruit development. We analysed global transcriptome dynamics using PCC and PCA analyses and assessed DETs for three different genotypes during four fruit developmental stages. Our results suggest that transcriptomic activity is more similar within genotypes than within development stages. The transcripts obtained at S1 confirm the active PA biosynthesis at early fruit developmental stages in all three genotypes. Transcripts generated during S4 are congruent with fruit maturation process during this stage. WGCNA unraveled the PA-associated regulatory networks and 20 crucial hub TFs within the metabolic networks. Cluster_78388 (SBP protein), Cluster_63454 (bZIP TF), and Cluster_66595 (MYB protein) are the candidate PA regulators in Chinese genotypes. This study provides a global understanding of transcriptome dynamics across three persimmon genotypes throughout fruit development, and elucidates the regulatory networks underlying specific PA accumulation patterns.

## Methods

### Plant material and RNA extraction

The persimmon genotypes (*Diospyros kaki* Thunb.) LT, MP, and YH used for transcriptome sequencing were grown in the Persimmon Repository of Huazhong Agricultural University (Wuhan, China). The full bloom date in the year 2018 is 27^th^, April. And fruit samples of these genotypes were collected at 2.5, 10, 20, and 25 weeks after bloom in 2018. Each sample was composed of three biological replicates, and at least 5 fruits were sampled for each biological replicate. The flesh in equatorial plane was cut into pieces, frozen in liquid nitrogen immediately, and then stored in the -80 °C refrigerator for RNA extraction.

The total RNA of the fruit samples were isolated using TRIzol reagent (Invitrogen, CA, USA) according to the manufacturer’s protocol. The quality, quantity, and integrity of RNA were assessed by Agilent 2100 bioanalyzer (Agilent Technologies, CA, USA) and NanoDrop 2000 spectrophotometer (Thermo Fisher Scientific, Massachusetts, USA). All RNA showed RIN > 8.

### Measurement of soluble and insoluble PAs

A 1-g fruit sample was finely grinded into powder for PAs extraction using Folin-Ciocalteu method. The soluble PAs were extracted in 80% methanol solution at room temperature, then the precipitates were used for insoluble PAs extraction in 1% (v/v) HCl-methanol under 65 °C water bath. The detailed interpretation was described in previous study [60].

### PacBio Iso-seq library construction and sequencing

The high-quality total RNA samples from four stages of LT fruits were pooled in equal amount to generate the full-length cDNA libraries for Iso-seq. The Iso-seq template was prepared following the protocol of Iso-Seq Template Preparation for Sequel Systems. The first cDNA strand was synthesised by Clontech SMARTer PCR cDNA Synthesis Kit (Takara Bio, Shiga, Japan). The CDS Primer IIA was first annealed to the polyA + tail of transcripts, followed by first-strand synthesis with SMARTScribe Reverse Transcriptase (Takara Bio, Shiga, Japan). Afterward, the double-stranded cDNA was prepared by large-scale PCR with Clontech PrimeSTAR GXL DNA Polymerase (Takara Bio, Shiga, Japan) and 5’ PCR Primer IIA (5’- AAGCAGTGGTATCAACGCAGAGTAC-3’). Two size-fractionated libraries of 0–5 kb and 4.5–10 kb were constructed to avoid the loading bias by partitioning the cDNAs into two size-ranges 0–5 kb and 4.5–10 kb using BluePippin Size-Selection System (Sage Science, Massachusetts, USA). The size selected cDNA products were applied to construct SMRTbell libraries by SMRTBell Template Prep Kit referring to manufacturer’s protocol (PacBio, CA, USA). SMRTbell sequencing libraries were bound to polymerases by using Sequel Binding Kit 2.1 and Primers V3 (PacBio, CA, USA). Afterward, the polymerase-template complexes were bound to MagBeads with the PacBio MagBead Binding Kit or diffusion loading (PacBio, CA, USA). Sequencing reactions were performed by PacBio Sequel sequencer (BGI-Shenzhen, China).

### PacBio Iso-Seq data analysis

Raw sequencing data generated from PacBio sequel were processed according to the IsoSeq protocol through SMRT analysis software (v 2.3.0) [61]. The ROIs were obtained from each library by filtering raw polymerase reads with the criteria of minimum full pass of 0 and minimum read score of 0.75. The full-length ROIs were determined by the presence of 5’, 3’ primers and the polyA tail preceding the 3’ primer. Non-full-length ROIs were defined by detection of the 5’ or 3’ primer. The full-length and non-full-length ROIs with the length less than 300 bp were removed in further analysis. To improve the accuracy of isoforms, the full-length and non-full-length transcripts were clustered by Interative clustering and error correction algorithm and then polished using Quiver quality-aware algorithm to generate hq isoforms. The hq isoforms with the accuracy > 0.95 from each library were merged together and the redundant sequences were removed by CD-HIT [62]. The non-redundant isoforms were further corrected using Illumina short reads by Proovread program [63]. The integrity of the full-length transcriptome was assessed by BUSCO [40]. All the full-length transcripts were aligned against the following databases for functional annotation: NR [64], NT, SwissProt [65], KEGG [66], KOG [67], Pfam [68], and GO databases [69]. The alignment was conducted by Blastn (v 2.2.23) [70] for NT annotation, Diamond (v 0.8.31) [71] for NR, KOG, KEGG, and SwissProt annotations, and Blast2GO (v 2.5.0) [72] for GO annotation based on NR annotation.

### CDS, SSR, TF, and lncRNA prediction

CDSs were determined by retaining the open reading frames (ORFs) showing homology with SwissProt and Pfam databases. The CDS of the transcripts were predicted using TransDecoder package (v 3.0.1) [73], and the candidate coding regions with the largest ORF were selected to search against the SwissProt database using Diamond Blastp (v0.8.31) [71]. Afterward, the results were used to detect the Pfam domain by Hmmscan software (HMMER, v 3.0) [74]. The ORFs with the Blastp hits and Pfam domain were retained in TransDecoder. Predict module.

The MISA program (v1.0) was used to detect SSRs of the reference transcriptome [75]. At least twelve repeats for mono-nucletide SSR, six repeats for di-nucletide SSR, five repeats for tri-nucletide SSR, four repeats for quad-nucletide SSR, three repeats for penta-nucletide SSR, and two repeats for hexa-nucletide SSR were considered as SSRs. And a sequence with two or more SSRs with the interrupted length less than 100 bp was identified as SSRs present in compound formation.

The TFs were determined by PlnTFDB database [76]. The ORFs of the isoforms were obtained by getorf package (EMBOSS: 6.5.7.0) [77], then compared against the PlntTFDB database to identify the TFs and assigned them to different families by hmmseach software (HMMER, v 3.0) [74].

The coding and noncoding potential of the full-length transcripts were examined by coding potential calculator (CPC) [78], Coding-Non-Coding Index (CNCI) [79], txCdsPredict [80], and Pfam analyses [81]. The noncoding sequences were defined by the criteria: CPC and CNCI scores were less than 0, txCdsPredict score was less than 500, and no protein-coding domain were detected by Pfam database. The sequences at least meeting three of the above four criteria were determined as lncRNAs.

### Illumina RNA-seq library preparation and sequencing

The 36 high-quality total RNA samples from the three genotypes at four developmental stages were used for libraries construction. The mRNA was purified using oligo (dT)-attached magnetic beads. Purified mRNA was randomly fragmented into pieces with fragmentation buffer under appropriate temperature. First-strand cDNA was amplified by First Strand reaction system, and the second-strand cDNA was generated, subsequently. A-Tailing Mix and RNA Index Adapters were added by incubating to end repair. The cDNA fragments with adapters were amplified by PCR, and the products were purified by Ampure XP Beads (Beckman Coulter, CA, USA). Then cDNA libraries were assessed using Agilent 2100 bioanalyzer (Agilent Technologies, CA, USA). Thereafter, the libraries were sequenced on Illumina Hiseq X Ten platform (Illumina, CA, USA) at BGI Genomics Ltd (BGI-ShenZhen, China).

### Illumina RNA-seq data analysis

The raw data of Illumina sequencing were assessed by SOAPnuke software (v 1.4.0) [82], and the adapters, the reads whose unknown base (‘N’ base) ratio is more than 5%, and the reads whose low-quality base ratio (base quality ≤ 5) is more than 20%, were removed by trimmomatic package (v 0.36) [83]. The clean reads were mapped to the PacBio full-length reference transcriptome using Bowtie2 program (v 2.2.5) [84], the number of reads for each transcript were calculated and normalised to FPKM by RSEM software (v 1.2.8) [85]. A transcript with the FPKM value ≥ 0.5 in at least one sample was considered as an expressed transcript. The DETs were determined by DEGseq2 software with the criteria of fold change ≥ 2 and the Qvalue (adjusted Pvalue) ≤ 0.001 [86].

PCC analysis of replicates was used to quantify the reproducibility of the transcriptome data of different developmental stages. Replicate LTS1_1 and MPS3_2 were removed for further analysis due to low correlation coefficient with their corresponding triplicates. Then the PCC plot of the retaining 34 samples is depicted in Additional file 2: Figure S6. Prcomp and cor.test in R were employed for PCA and PCC analyses, respectively.

### Identification of the SS transcripts

The SS transcripts showing preferential expression at a particular developmental stage were identified by comparing the expression of a transcript at a given stage with its maximal expression at the other stages. The transcripts with FPKM value ≥ 2 in all the replicates were used as input data to calculate the SS scores using rsgcc (v 1.0.6) package [49, 87]. The higher SS score of a transcript at a stage signifies the more specific expression of the transcript at that stage. We defined that a transcript with SS score ≥ 0.5 at a particular stage was SS transcript at that stage.

### Weighted gene co-expression network analysis

The transcripts were enriched and the co-expression networks with highly connected expression patterns were constructed using WGCNA. The co-expression modules were constructed using the one-step network construction with default settings following the tutorial [88]. The transcripts with FPKM ≥ 2 across all samples were used to calculate the coefficient of variance, and the top 8,000 transcripts with the highest coefficient of variance were subjected to WGCNA analysis. A matrix of pairwise PCCs between all pairs of the genes was generated based on log2 (1 + FPKM) values, and then transformed into an adjacency matrix using the formula: connection strength (adjacency value) =|(1 + correlation)/2| Power. The power was the soft threshold for correlation matrix, which was produced by function pickSoftThreshold. And it was set to 9 to make the network fit scale-free topology (Additional file 2: Figure S7). The adjacency matrix was converted to topological overlap (TO) matrix by TOM similarity algorithm, and the transcripts were hierarchically clustered depending on TO similarity. The first principal component of each module was represented by the module eigengene which can be considered as representative of expression profile in the module. And the module-sample associations were calculated using eigengene expression value and the PA content by cor function. GS is any quantitative measure that specifies how biologically significant a gene is. The higher the absolute value of GS of a gene, the more biologically significant this gene is. Intramodular connectivity measures how connected a given gene is to biologically interesting modules. The transcripts in the interesting module with high GS and intramodular connectivity were identified as candidate transcripts, based on the criteria of abs (GS) > 0.6 & abs (kME) > 0.8. The correlation network of MEblue and MEbrown modules were visualized by Cytoscape (v 3.7.2) [89]. The co-expression transcripts and the related edges with the node-edge connectivity ≥ 0.43 for MEblue and 0.5 for MEbrown were depicted.

## Supplementary Information

**Additional file 1.**

**Additional file 2.**

**Additional file 3.**

**Additional file 4.**

**Additional file 5.**

**Additional file 6.**

## Data Availability

The RNA sequencing reads of two PacBio Iso-seq libraries and 34 Illumina RNA-seq libraries are available in the Sequence Read Archive database of NCBI (BioProject ID: PRJNA715943). And this Transcriptome Shotgun Assembly project (TSA) has been deposited at Genbank under the accession GJEX00000000. The version described in this paper is the first version, GJEX01000000 with 1 of the 96,463 sequences further removed by NCBI due to duplication. The datasets supporting the conclusions of this article are included within the article (and its additional files).

## Abbreviations

LT: Luotiantianshi
MP: Mopanshi
YH: Youhou
SSR: Simple sequence repeat
LncRNA: Long noncoding RNA
CDS: Protein-coding sequence
DET: Differentially expressed transcript
PCA: Principal component analysis
PCC: Pearson correlation coefficient
PAs: Proanthocyanidins
ANR: Anthocyanidin reductase
LAR: Leucoanthocyanidin reductase
PCNA: Pollination-constant nonastringent
C-PCNA: Chinese PCNA
J-PCNA: Japanese PCNA
TF: Transcription factor
PacBio: Pacific biosciences
S1: 2.5 Weeks after bloom
S2: 10 Weeks after bloom
S3: 20 Weeks after bloom
S4: 25 Weeks after bloom
ROIs: Reads of inserts
hq: High-quality
BUSCO: Benchmarking universal single-copy orthologs
NCBI: National center for biotechnology information
NR: NCBI non-redundant protein
NT: NCBI non-redundant nucleotides
KEGG: Kyoto encyclopedia of genes and genomes
KOG: EuKaryotic orthologous groups
Pfam: Protein family
GO: Gene ontology
CPC: Coding potential calculator
CNCI: Coding non coding index
SS: Stage-specific
WGCNA: Weighted gene coexpression network analysis
SBP: SQUAMOSA promoter-binding protein
FPKM: Fragments per kilobase of transcript per million mapped reads
USA: United states
CA: California
GS: Gene significance
