# Supplementary material for: Comparative transcriptome analysis reveals regulatory network and regulators associated with proanthocyanidin accumulation in persimmon
Source: BMC Plant Biol. 2021 Jul 29;21:356. doi: 10.1186/s12870-021-03133-z (PMC8323215; doi:10.1186/s12870-021-03133-z)
Supplement: Supplementary file 2 — Additional file 2. [file 12870_2021_3133_MOESM2_ESM.docx]

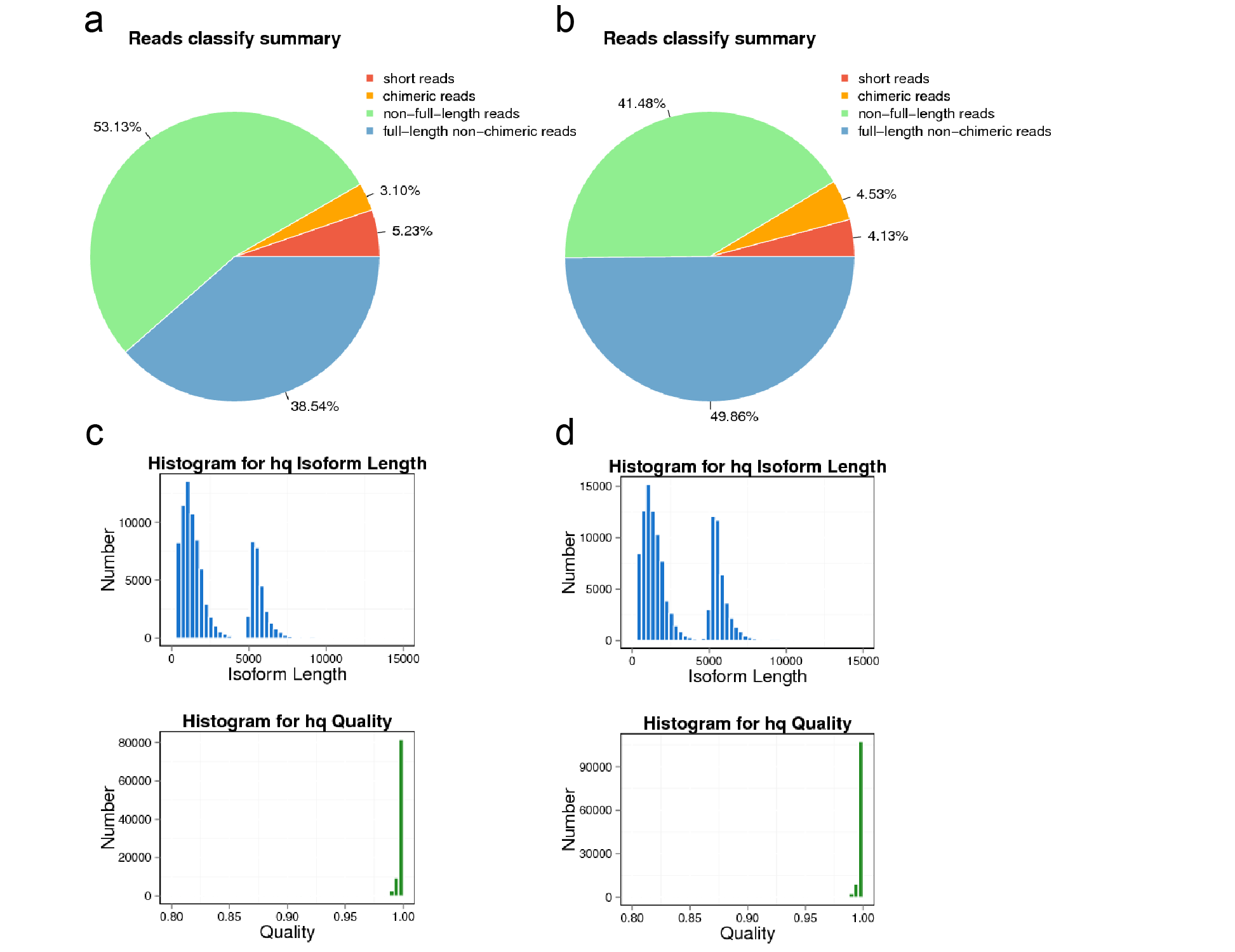


**Figure S1** Quality assessment of PacBio Iso-seq data. The pie chart of ROIs showing percentage of the four different categories, full-length non-chimeric reads, non-full-length reads, chimeric reads, and short reads in 0-5 Kb library (**a**) and 4.5-10 Kb (**b**) library, respectively. The histogram showing the length (upper panel) and quality score (below panel) distributions of hq isoforms in 0-5 Kb library (**c**) and 4.5-10 Kb (**d**) library, respectively.


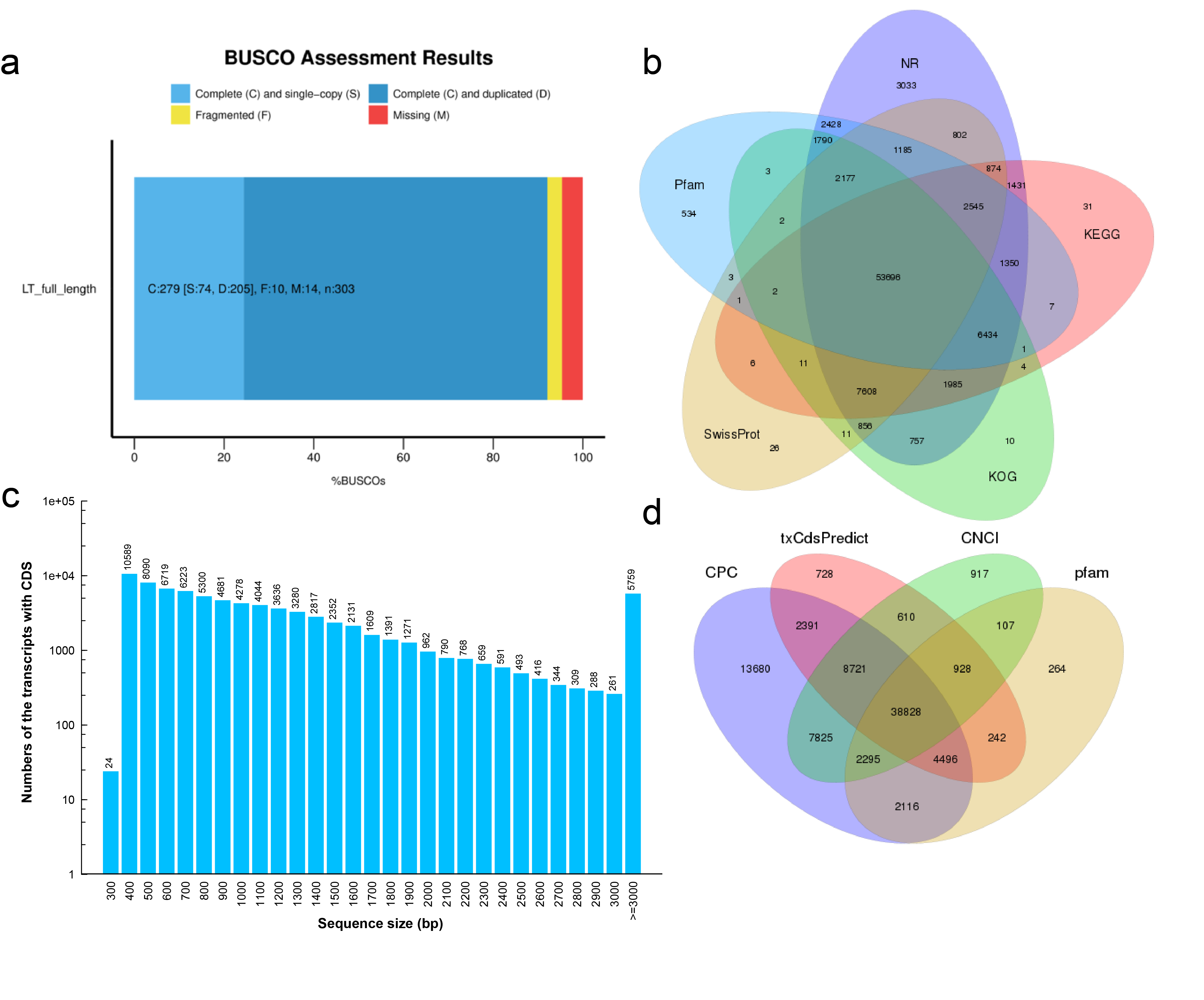


**Figure S2** Quality assessment, annotation, CDS, and lncRNA analyses of reference transcriptome. **a** The completeness of the reference transcriptome generated by PacBio platform was assessed by aligning to BUSCO database. **b** Venn diagram showing the number of transcripts annotated commonly or uniquely by NR, SwissProt, KEGG, Pfam, and KOG databases. **c** Length distribution of the predicted CDSs. **d** Venn diagram showing the number of lncRNAs identified commonly or uniquely by CPC, CNCI, txCdsPredict, and Pfam algorithms.


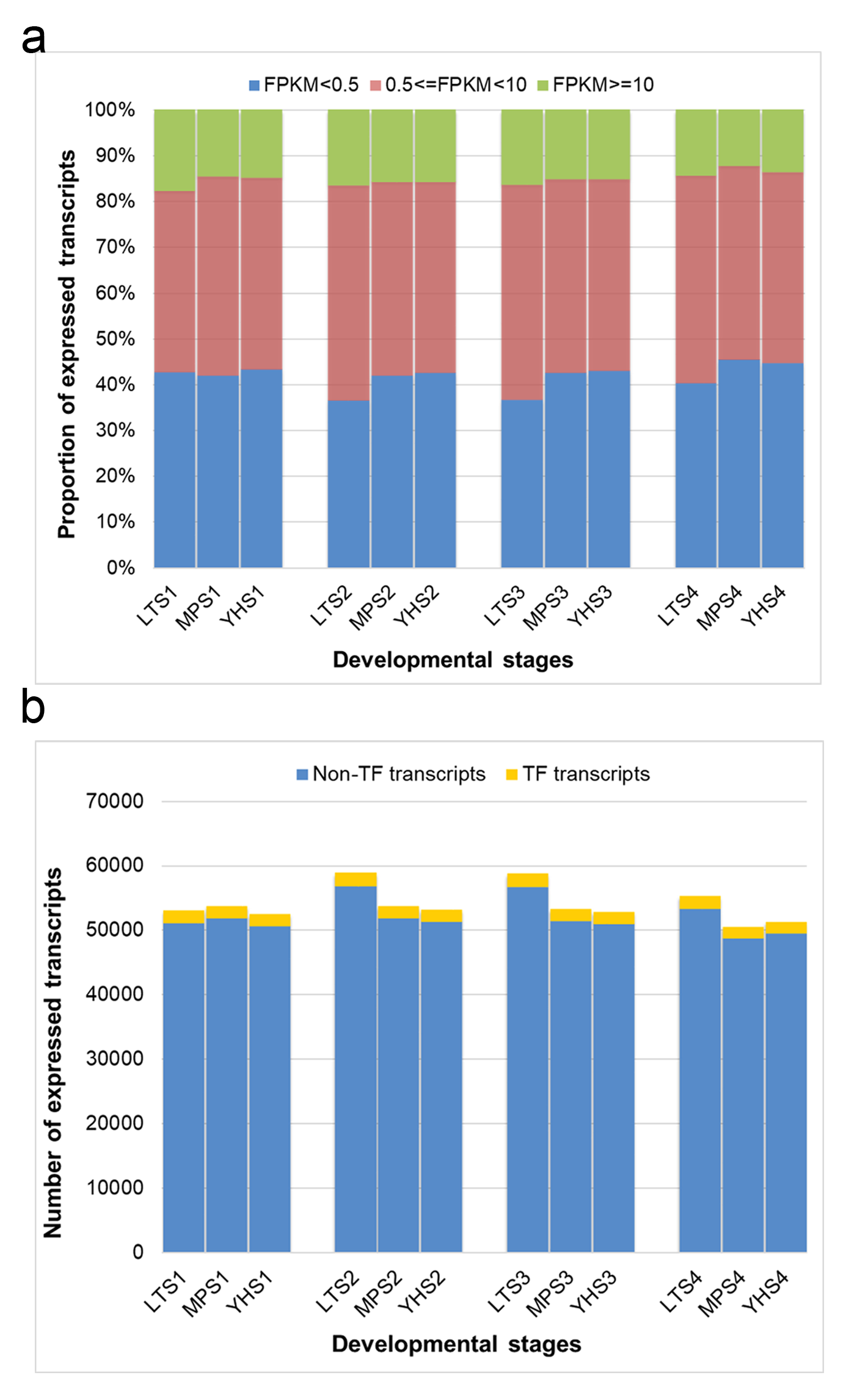


**Figure S3** Transcript expression profiling of LT, MP, and YH at four developmental stages. **a** Proportions of transcripts at different levels (based on FPKM value) in the three genotypes. **b** Numbers of expressed transcripts (FPKM ≥ 0.5) and TFs in the three genotypes.


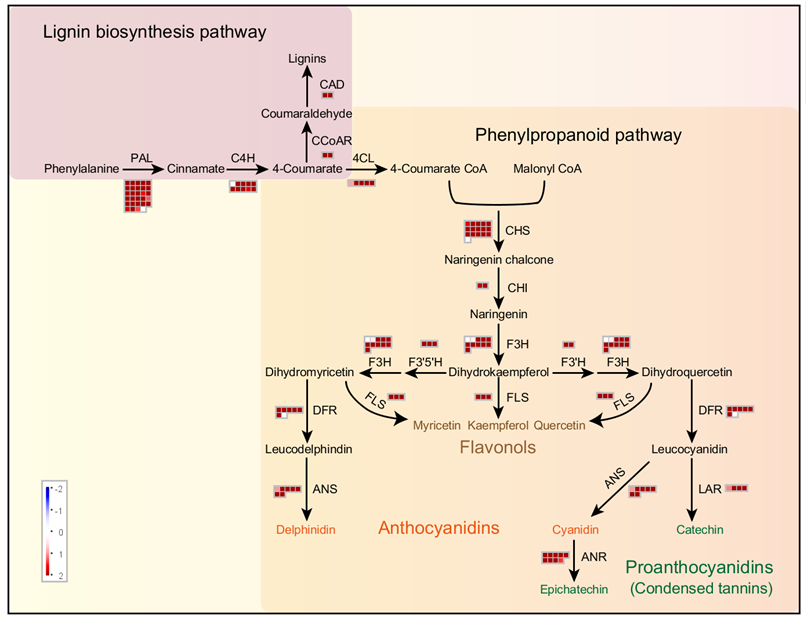


**Figure S4** Metabolic pathway analysis of the S1-specific transcripts with corresponding expression values at S1 in LT using MapMan tool. A cell represents a pathway biosynthetic transcript. Dark red and dark blue colours represent higher and lower expression level based on the expression level at S1 in LT genotype.


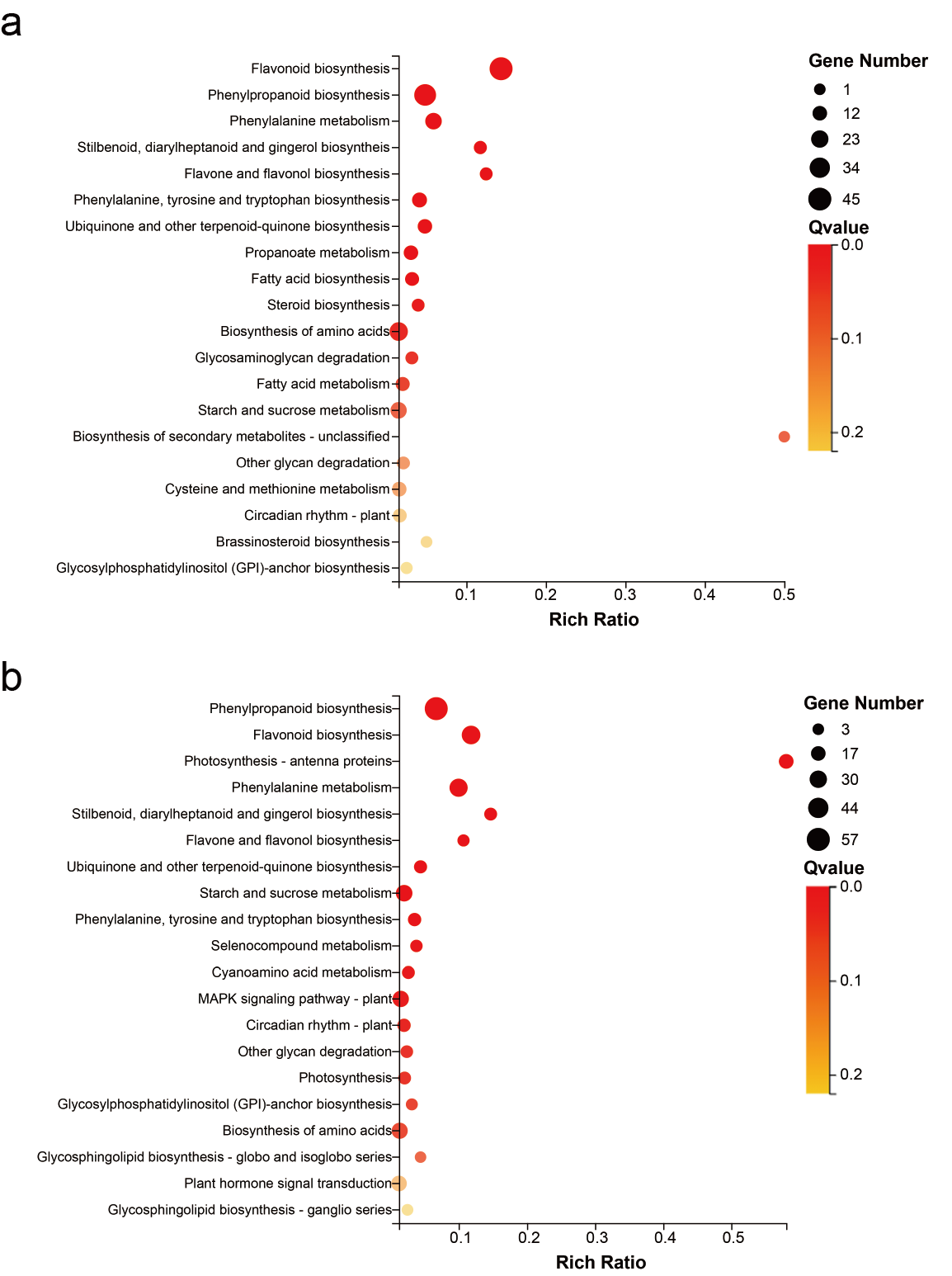


**Figure S5** KEGG enrichment analyses of the transcripts within MEbrown module (**a**) and MEblue module (**b**). The size of the circle represents the number of enriched transcripts. The colour represents the Qvalue (adjusted p-value).


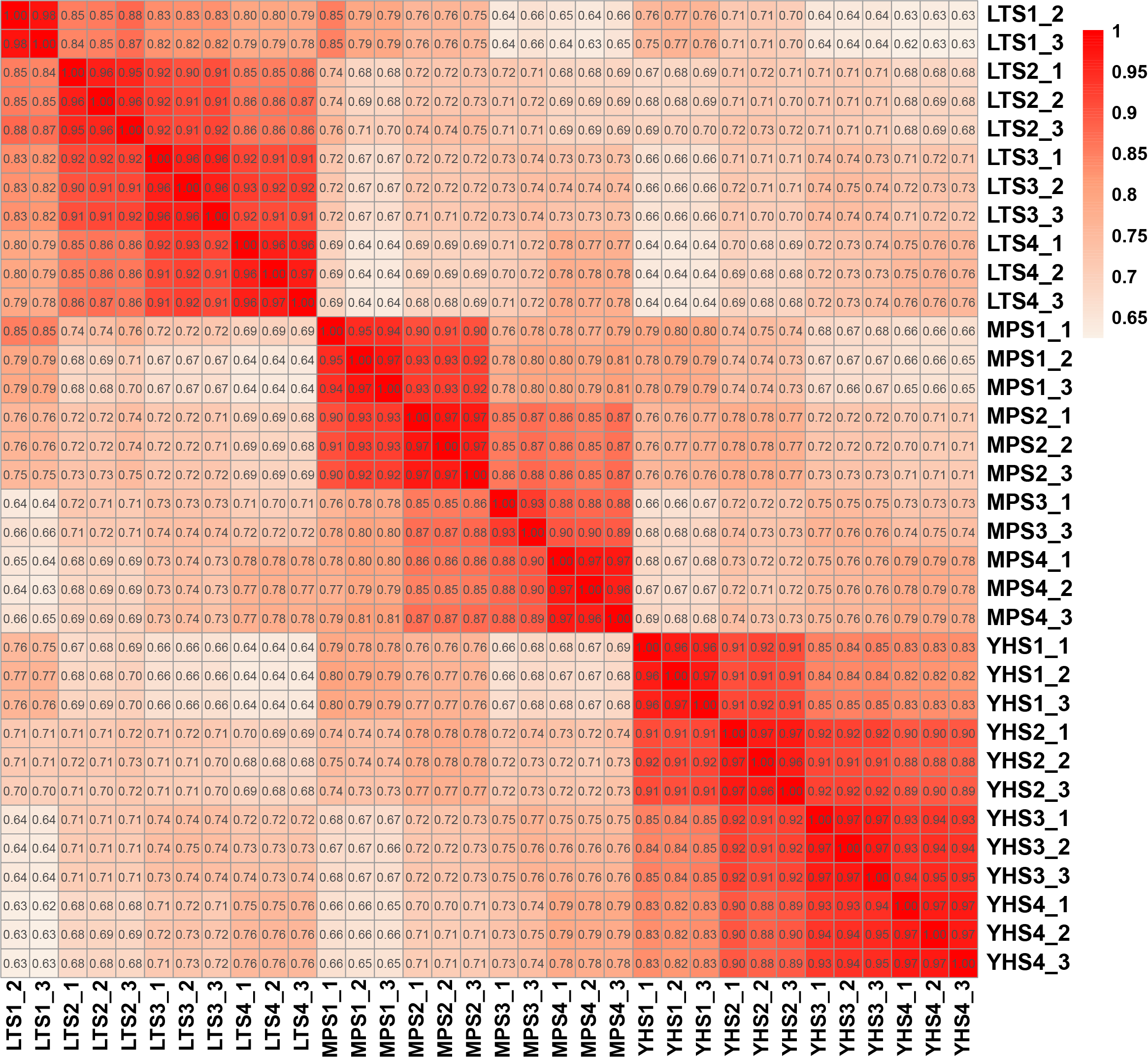


**Figure S6** PCC analysis of the biological replicates of LT, MP, and YH genotypes at four stages.


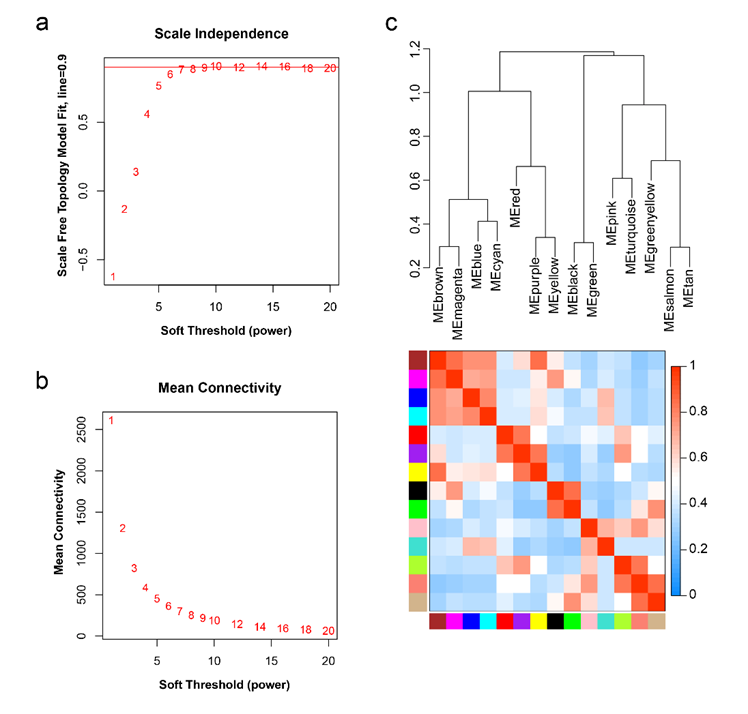


**Figure S7** Soft-thresholding powers selection for scale-free topology. **a** The scale-free fit index under incremental soft-thresholding power. **b** The mean connectivity under incremental soft-thresholding power. **c** Hierarchical clustering of 14 WGCNA co-expression modules.
